# Analyzing self-evaluation capacity scores related to infectious disease control in International Health Regulations during the first year of COVID-19 pandemic

**DOI:** 10.1038/s41598-022-19361-8

**Published:** 2022-09-02

**Authors:** Fauzi Budi Satria, Feng-Jen Tsai, Battsetseg Turbat

**Affiliations:** 1 Ph.D. Program in Global Health and Health Security, College of Public Health, Taipei Medical University, Taipei City, Taiwan; 2 Master Program in Global Health and Development, College of Public Health, Taipei Medical University, Taipei City, Taiwan

**Keywords:** Viral infection, Health policy

## Abstract

This study aimed to identify changes in the average score of countries' International Health Regulation (IHR) self-evaluation capacity (e-SPAR) in 2020 compared to 2019, and the factors associated with these changes. We collected the data from the World Health Organization (WHO) website in May 2021, then calculated the significant differences between the e-SPAR score in both years. Next, we conducted a test to identify the association between changes in member states' e-SPAR capacity scores and their COVID-19 case fatality rate (CFR), Human Development Index, Civil Liberties, and Government Effectiveness. The results showed that the average e-SPAR scores in 2020 were significantly higher than in 2019. Among the 154 countries, we included in this study, the scores of 98 countries increased in 2020, of which 37.75% were lower-middle-income countries. Meanwhile, among the 56 countries whose scores did not increase, 26 (46.42%) were high-income countries. The COVID-19 CFR was significantly associated with the changes in e-SPAR scores of 154 countries (p < 0.01), as well as the countries whose scores increased (p < 0.05). In conclusion, we consider e-SPAR to still be a useful tool to facilitate countries in monitoring their International Health Regulation (IHR) core capacity progress, especially in infectious disease control to prepare for future pandemics.

## Introduction

Emerging infectious diseases such as COVID-19 have become a significant health and security challenge for the world. After the Severe Acute Respiratory Syndrome (SARS) pandemic in 2003, International Health Regulation 2005 (IHR 2005) was adopted by the World Health Organization (WHO) to help countries in setting up their national preparedness for an efficient early alert and response system in handling public health events and emergencies that have the potential to cross borders^1^. One of the approaches adopted by IHR 2005 is to require member states to develop minimum core capacities for infectious disease control. To monitor progress, WHO requires member countries to conduct a self-evaluation of their IHR core capacity annually^2,3^. However, the involvement of member countries in responding to the requirement was initially unsatisfactory. Then, an external evaluation approach (Joint External Evaluation, JEE) was introduced and implemented in 2016 after several pandemics occurred globally from 2012 to 2015^2,4^.

Both self-and external-evaluation approaches have been proven to be accountable and effective in helping member countries increase their capacity to prepare for major public health threats^5–7^. A study using Infectious Disease Threat Events (IDTE) data between 2010 and 2016 showed that a 10% increase in IHR core capacity scores was associated with a 14% to 20% reduction in cross-border IDTE incidents globally^6^. Moreover, in another study that focused on the relationship between countries’ IHR core capacity score and their infectious disease control outcomes, the results showed that countries with low average scores on IHR State Party Self-Assessment Annual Report (SPAR) in 2016 and 2017 had a significantly higher risk of having bad infectious disease outcomes than countries with high IHR average scores^3^. However, in another study, it was found that some countries’ self-reported scores were consistently 1 to 1.5 points higher than externally assessed scores in the same year. This finding reflects the phenomenon that countries tend to over-score their capacity, especially countries that lack transparency^8^.

In the context of the COVID-19 pandemic, a paper published in the early stage of the global pandemic claimed that countries labeled with strong operational preparedness capacities on e-SPAR (Electronic State Party Self-Assessment Annual Report) should have the capacity to respond to the COVID-19 pandemic effectively^9^. However, although the results of many studies predict the effectiveness of SPAR in measuring the capacity of countries to deal with the crisis, the world is not yet recovered from the COVID-19 pandemic. Currently, there have been more than 250 million cases and 5 million deaths globally, including in the Americas and European region^10^. A more recent study evaluating the relationship between country preparedness capacity presented by e-SPAR and country COVID-19 control outcomes demonstrated the limited effectiveness of such scores even before COVID-19^11^.With the hypothesis that countries’ e-SPAR scores will decrease after the COVID-19 pandemic, we conducted this study to determine the changes in scores in 2019 and 2020, as well as the factor associated with these score changes.

## Results

### The changes in e-SPAR capacity scores between 2019 and 2020

**Figure 1:**
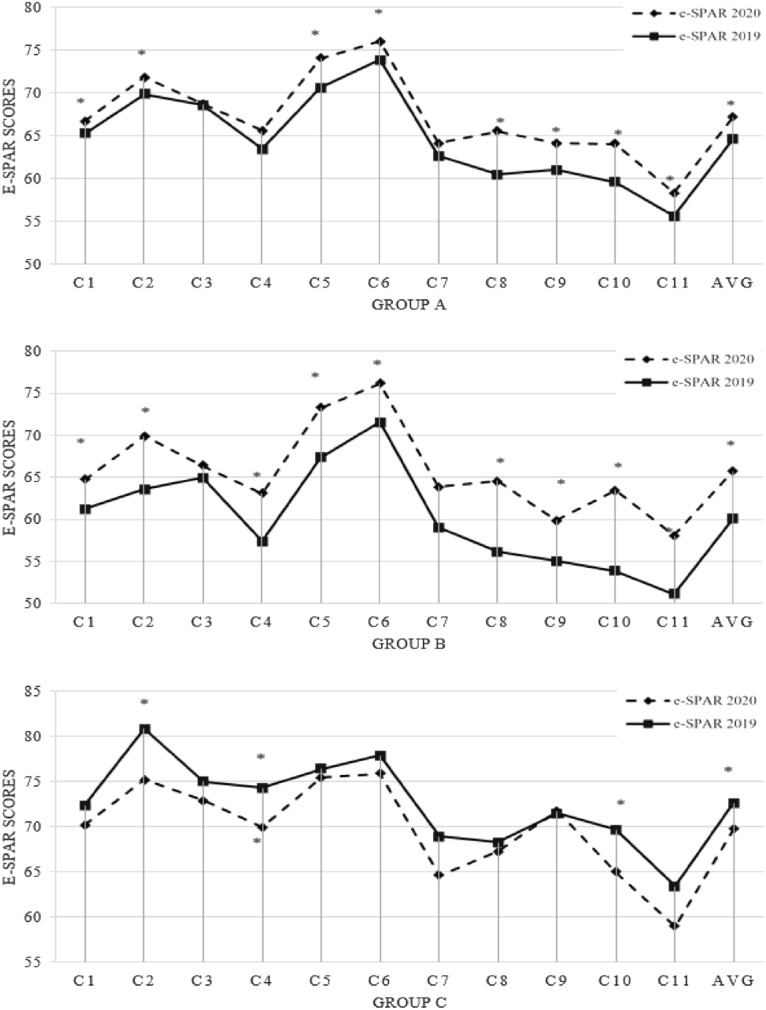
The countries’ e-SPAR capacity scores in 2020 and 2019; asterisk: capacity with significant change (p < 0.05). Group A: 154 countries, Group B: 98 countries whose score increased, Group C: 56 countries whose score did not increase . Capacity: Legislation and financing (C1), IHR coordination and National IHR focal point function (C2), Zoonotic events and the human-animal interface (C3), Food safety (C4), Laboratory (C5), Surveillance (C6), Human Resources (C7), National Health Emergency Framework (C8), Health Service Provision (C9), Risk communication (C10), Points of entry (C11), Average of 11 capacities (AVG). This figure showed the countries’ e-SPAR capacity scores in 2020 and 2019. Among all 154 countries (group A), the average of 11 e-SPAR capacity scores as well as the scores for each capacity in 2020 were higher than their respective scores in 2019. From 2019 to 2020 the scores increased from 0.13 to 5.04. From the test, it’s known that within this group, capacities about Zoonotic events and the human-animal interface, Food Safety, and Human Resources (p > 0.05) were the three capacities that were significantly different. Next, among 98 countries whose scores increased (group B), the test result showed that the average score of the 11 capacities increased significantly from 2019 to 2020. Risk communication, National Health Emergency Framework, and Ports of entry were the 3 capacities that mostly increased (p < 0.05). In this group, the capacity of Zoonotic events and the human-animal interface was the only capacity that was not significantly changed during the pandemic (p > 0.05). Meanwhile, among 56 countries whose scores did not improve (group C), the test result showed the average score of the 11 capacities decreased significantly, as well as the capacity for National IHR focal point function, Food Safety, and Risk Communication (p < 0.05). Meanwhile, other capacities were not significantly changed during the first year of the COVID-19 pandemic, including capacity related to zoonotic disease control.

The 2019 and 2020 e-SPAR scores can be seen in Fig. 1. Among all 154 countries (group A), the average of 11 e-SPAR capacity scores as well as the scores for each capacity in 2020 were higher than their respective scores in 2019. From 2019 to 2020 the scores increased from 0.13 to 5.04. From the Wilcoxon signed-rank test, the average score of 11 capacities and the scores of all individual items in 2020 were significantly different from the score in 2019, except for capacities related to Zoonotic events and the human-animal interface, Food Safety, and Human Resources (p > 0.05). Next, among the 98 countries whose scores increased (group B), the Wilcoxon signed-rank test result showed that the average score of the 11 capacities increased significantly from 2019 to 2020. Risk communication, National Health Emergency Framework, and Ports of entry were the three capacities that mostly increased (p < 0.05). However, the capacity of Zoonotic events and the human-animal interface was the only capacity that was not significantly changed during the pandemic (p > 0.05). Meanwhile, among the 56 countries whose scores did not improve (group C), the Wilcoxon signed-rank test results showed the average score of the 11 capacities decreased significantly, as well as the capacity for National IHR focal point function, Food Safety, and Risk Communication (p < 0.05).

Description of COVID-19 CFR, countries’ income level, HDI, CL, and GE among overall countries (group A), countries whose scores increased (group B) and not increased (group C) are shown in Table 1. Based on country income level, among the countries that experienced an increase in scores, the majority were lower-middle-income countries (LMICs) (37.75%), while only 18.4% of high-income countries (HICs) and low-income countries (LICs) showed an increase in scores. Meanwhile, HICs made up the majority of countries whose scores did not increase (46.43%). Similar to the distribution of countries’ income levels, countries with medium development status were the group that mostly (28.57%) experienced an increase in e-SPAR scores during the pandemic. Meanwhile, countries with very high development status made up the majority of countries whose scores​​ did not increase (55.36%) in 2020 compared to the previous year.

**Table 1:** Description of COVID-19 CFR, countries’ income level, HDI, CL, and GE among overall countries (group A), countries whose scores increased (group B) and not increased (group C).

	Overall	Scores increased	Scores did not increase	Chi-square		
	n = 154 (%)	n = 98 (%)	n = 56 (%)	X^2^	*p-*value	Phi/Cramer’s* V*
**CFR of COVID-19**				0.00	1.00	0.00
Low	99 (64.29)	63 (64.29)	36 (64.29)			
High	55 (35.71)	35 (35.71)	20 (35.71)			
**Countries’ income level**				16.79	0.00	0.33
LICs	24 (15.58)	18 (18.37)	6 (10.71)			
LMICs	46 (29.87)	37 (37.75)	9 (16.07)			
HMICs	40 (25.97)	25 (25.51)	15 (26.79)			
HICs	44 (28.57)	18 (18.37)	26 (46.43)			
**Human development index**				20.89	0.00	0.37
Low	32 (20.78)	24 (24.49)	8 (14.29)			
Medium	32 (20.78)	28 (28.57)	4 (7.14)			
High	37 (24.03)	24 (24.49)	13 (23.21)			
Very high	53 (34.41)	22 (22.45)	31 (55.36)			
**Civil liberties**				2.75	0.25	0.13
Not free	41 (26.62)	26 (26.53)	15 (26.79)			
Partially free	53 (34.41)	38 (38.77)	16 (34.57)			
Free	60 (38.96)	34 (34.69)	26 (46.43)			
**Government effectiveness**				16.76	0.00	0.33
Weak	83 (53.90)	65 (66.33)	18 (32.14)			
Strong	71 (46.10)	33 (33.67)	38 (67.86)			

This table describes the COVID-19 CFR, countries’ income level, HDI, CL, and GE among overall countries (group A), countries whose scores increased (group B) and not increased (group C). Based on country income level, among the countries that experienced an increase in scores, the majority were lower-middle-income countries (LMICs) (37.75%), while the high-income countries (HICs) and low-income countries (LICs) were the least with 18.37% each. On the contrary, HICs were the majority among countries whose scores did not increase (46.43%). Similar to the distribution of income country level, countries with medium development status were the group that mostly (28.57%) experienced an increase in e-SPAR scores during the pandemic. Meanwhile, countries with very high development status were the majority in the group of countries whose scores did not increase (55.36%) in 2020 compared to the previous year. Next, for civil liberties status, most countries within the group of countries whose scores increased during the pandemic were “partially free” countries (38.77%), followed by “free” countries (34.69%), and the least was “not free” countries (26.53%). Meanwhile, among the countries whose scores did not increase during the pandemic, most of them were “free” countries (46.43%). Then, for government effectiveness, most of the countries whose scores increased during the pandemic had weak GE (66.33%) while most of the countries whose scores did not increase had a strong GE (67.86%). Meanwhile, for CFR of COVID-19, 64.29% of the countries in each group had low CFR.

When assessed in terms of Civil Liberties, most countries whose scores increased during the pandemic were “partially free” countries (38.77%), followed by “free” countries (34.69%), and finally “not free” countries (26.53%). Meanwhile, most of the countries whose scores did not increase during the pandemic were “free” countries (46.43%). Most of the countries whose scores increased during the pandemic had “weak” Government Effectiveness (GE) (66.33%) while most of the countries whose scores did not increase had a “strong” GE (67.86%). Meanwhile, the COVID-19 Case Fatality Rate was “low” for 64.29% of the countries in each group.

### Factors associated with changes in the e-SPAR scores

Associations between countries’ changes in e-SPAR scores with HDI, CL, GE, and CFR are shown in Table 2. Models 1 and 2 were able to significantly described the changes in e-SPAR scores among 154 countries (R^2^ = 0.14, Adjusted R^2^ = 0.12, p < 0.01) and among 98 countries whose scores increased (R^2^ = 0.20, Adjusted R^2^ = 0.17, p < 0.01). Meanwhile, Model 3 was not able to significantly describe the changes in e-SPAR scores among 56 countries whose scores did not increase (R^2^ = 0.06, Adjusted R^2^ = 0.01, p = 0.05). In Model 1; of the four variables, we considered, the CFR of COVID-19 (B = −0.61, p < 0.05) and GE (B = −2.08, p = 0.05) were the factors significantly associated with changes in e-SPAR scores during the pandemic in 154 countries. In addition, while in Model 2 the CFR of COVID-19 (B = -0.54, p < 0.05) was the only variable that significantly associated with the changes in e-SPAR scores, none of the variables in Model 3 were individually associated with the changes in e-SPAR scores.

**Table 2:** The multiple regression analysis results for group A (Model 1), group B (Model 2), and group C (Model 3).

	Unstandardized coefficient		Standardized coefficient	t	p	VIF
	B	Std. error	Beta			
**Model 1**						
**e-SPAR scores changes**						
HDI	−5.33	5.89	−0.14	−0.90	0.37	3.91
CL	0.03	0.02	0.16	1.51	0.13	2.03
GE	−2.08	1.05	−0.34	−1.98	0.04*	5.19
CFR	−0.61	0.23	−0.21	−2.64	0.01*	1.09
**Model 2**						
**e-SPAR scores changes**						
HDI	−9.60	5.29	−0.33	−1.81	0.07	3.81
CL	−0.811	1.00	−0.17	−0.81	0.42	5.24
GE	0.02	0.02	0.12	0.88	0.38	2.03
CFR	−0.54	0.27	−0.19	−2.04	0.04*	1.06
**Model 3**						
**e-SPAR scores changes**						
HDI	8.29	8.21	0.26	1.01	0.32	3.62
CL	−0.49	1.36	−0.10	−0.36	0.72	4.49
GE	0.01	0.03	0.06	0.29	0.77	2.00
CFR	−0.13	0.26	−0.08	−0.52	0.61	1.22

From the table, it can be seen that Models 1 and 2 were able to significantly describe the changes in e-SPAR scores among 154 countries (constant = 5.55, F = 5.21, p < 0.01, R^2^ = 0.14, adjusted R^2^ = 0.12) and among 98 countries whose scores increased (constant = 12.08, F = 5.91, p < 0.01, R^2^ = 0.20, adjusted R^2^ = 0.17), while Model 3 was not (constant = −9.33, F = 0.84, p < 0.05, R^2^ = 0.06, Adjusted R^2^ = 0.01). In Model 1, of the four variables, the CFR of COVID-19 (p < 0.05) and GE (p < 0.05) were the factors that were significantly associated with the changes in e-SPAR scores during the pandemic in 154 countries. Meanwhile, in Model 2, the CFR of COVID-19 (p < 0.05) was the only variable that was significantly associated with the changes in e-SPAR scores during the pandemic among countries whose scores increased.

## Discussion

The results of this study indicate that the average e-SPAR score of 154 countries in 2020 was significantly different from the score in 2019. There were 98 countries (63.63%) that experienced an increase in e-SPAR scores in 2020, while 56 countries (36.36%) experienced a decrease in e-SPAR scores. Among those whose scores increased, 63.26% were middle-income countries. Risk communication, National Health Emergency Framework, and Ports of entry were the 3 capacities that mostly increased while Zoonotic events and the human-animal interface was the only capacity that did not increase significantly. Meanwhile, among countries whose scores did not increase, most (46.43%) were high-income countries. IHR Coordination, Risk Communication, and Food Safety were the capacities that were significantly decreased in this group.

The results show that e-SPAR is an effective tool for countries to monitor the progress of their IHR core capacities^12^. The decrease in e-SPAR scores among high-income countries might illustrate how they were using the pandemic as an opportunity to take a more rigorous approach to re-evaluate their capacity to be more accurate. Meanwhile, the increase in e-SPAR scores of low- and lower-middle-income countries during the pandemic might indicate their unpreparedness before the COVID-19 pandemic. In these countries, limited resources may have been a main factor for this unpreparedness as countries have had to prioritize other expenditures for their development^13,14^. However, COVID-19 may have provided them with an opportunity to allocate more resources to enhance their capacity in infectious diseases which then increased their IHR capacity scores in the first year of the pandemic.

Furthermore, our results showed that the capacity associated with zoonotic disease control was the only capacity whose scores did not change significantly in both score-increased and score-not-increased countries. This finding seems to show that the world is not ready to face a pandemic in the future, especially if it is a zoonotic disease. This is supported by many references showing that zoonotic diseases are major threats in the future^15,16^. Reported showed that most major public health events considered the Public Health Emergencies of International Concern (PHEIC) were caused by pathogens that originated from animals^15^. Worse yet, it is stated that most zoonotic disease-causing agents have the potential to be used as biological weapons for bioterrorism purposes^16^. Therefore, the approach taken by WHO in collaboration with organizations in various sectors such as Food and Agriculture Organization (FAO) and OIE (Office International des Epizooties/ the World Organization for Animal Health) to comprehensively address health problems through a One Health approach should be strengthened^17^.

Multiple regression analysis identifies COVID-19 CFR and GE as the two factors that are significantly associated with the changes in the e-SPAR scores of 154 countries. Several studies have also shown that countries with better government effectiveness scores, COVID-19 test numbers, and higher numbers of hospital beds^18^ had lower COVID-19 mortality rates^19,20^. These findings showed that though a government may be severely hit by COVID-19, they can still implement relevant policies with rationale. Therefore, although the COVID-19 pandemic caused great suffering, this crisis served as an opportunity for countries to better evaluate their capacity. Although the e-SPAR score was imprecise in predicting countries’ control outcomes, it is still useful in facilitating countries to monitor and improve the progress of their awareness and preparedness for future pandemics^21^.

Furthermore, the result of Model 2 showed that CFR was the only factor associated with the changes in e-SPAR scores in 2020 compared to 2019 (p < 0.05). While most of the countries with increased scores were middle-income countries, financial assistance may also have been the reason for the increase in their e-SPAR scores. Data showing the recipients of COVID-19 aid funds suggests that countries with a more severe COVID-19 burden received greater funds^22,23^. This is supported by our results showing a significant association between countries’ CFR of COVID-19 and their increase in e-SPAR scores. However, further research is recommended to better understand the reason behind this phenomenon.

Even though the pandemic is still ongoing, this study aims to highlight these important findings. In the first year of the COVID-19 pandemic, while countries worldwide struggled to control and mitigate the pandemic, our research assessed countries’ self-evaluation during a real crisis. Especially, as it is known that crisis of this scale does not occur regularly. A limitation of this study is that the changes in e-SPAR scores may have been under-scored as countries may have reported their e-SPAR scores only in the very early stage of the COVID-19 pandemic. These early e-SPAR scores may not be a true reflection of those countries’ self-evaluation of their IHR capacity during the actual pandemic. Furthermore, at the outset of this study in early 2021, GNI 2020 data was not yet available. However, 2019 data was used to illustrate how pre-pandemic economic situations of countries could affect outcomes in controlling COVID-19. In addition, regarding the linear regression models, factors such as the number of donors, amount of funding received, or budget allocated by countries during the crisis to control the pandemic could also be the factors associated with the e-SPAR changes but were not considered in this study. Finally, the design of this study only allows the results to be considered as an association rather than a causal relationship.

## Conclusion

The average of 11 e-SPAR capacity scores during the first year of the COVID-19 pandemic in 2020 was significantly different from the respective score in 2019. The lower-middle-income countries made up the majority of the countries whose e-SPAR scores increased, while high-income countries made the majority in the group whose scores did not increase. Our study results showed that CFR of COVID-19 was significantly associated with the changes in e-SPAR scores of 154 countries and 98 countries whose e-SPAR scores increased, but not countries whose e-SPAR scores did not increase. In conclusion, we consider that e-SPAR is an effective tool for countries to monitor the progress of their IHR core capacities.

## Methods

### Study framework

The framework of this study was adopted from the Systemic Rapid Assessment (SYSRA) toolkit. We adopted SYSRA because of its coherence to the requirement for countries in implementing the IHR. In SYSRA, there are two types of assessment; horizontal and vertical. The “horizontal assessment” analyzes the health system within which the infectious disease program is embedded from a variety of perspectives. While, the second element, the “vertical assessment” is used to assess the infectious disease-specific component. Thus, both elements index the external environment (political, socio-demographic, economic) and need assessment (morbidity, mortality of the disease) as a consideration in assessing infectious diseases control programs^2,24,25^.

### Self-evaluation capacity (e-SPAR) scores related to infectious disease control

To identify the changes in countries’ self-evaluation capacity (e-SPAR) scores related to infectious disease control during the first year of the COVID-19 pandemic, we calculated the absolute difference between the score of IHR e-SPAR in 2019 and 2020. We collected this data from the WHO website in May 2021. There was a total of 13 items in the e-SPAR including Legislation and financing, IHR coordination and National IHR focal point function, Zoonotic events and the human-animal interface, Food Safety, Laboratory, Surveillance, Human Resources, National Health Emergency Framework, Health Service Provision, Risk communication, Points of entry, Chemical events and Radiation emergencies^26^. Since our study focused on infectious diseases control, we excluded Chemical events and Radiation emergencies capacity scores for analysis and only used 11 of 13 items in e-SPAR.

### Case fatality rates (CFR) of COVID-19

We used deaths instead of cases to reduce bias in the data we analyzed. This is because there are 3 levels used in the diagnosis of COVID-19 cases; suspected, probable, and confirmed cases. Thus, data on the number of confirmed cases of COVID-19 will fluctuate and be unstable due to changes in the diagnosis status of a patient^27^. Additionally, in reporting deaths due to COVID-19, only deaths that were confirmed to be caused by COVID-19 were reported^27^. As the pandemic is still ongoing, we used the Case Fatality Rate (CFR) of COVID-19 data up to March 31st 2021 to represent the CFR of COVID-19 in one year. We gathered COVID-19’s CFR until March 31st 2021 from the “Our World in Data” website^27^. While until late 2021 the CFR of COVID-19 was ranging from 2 to 3% globally^28,29^, in this study, we used 2.08% as the cut-off point to classify countries into high CFR or low CFR groups. This number resulted from calculating the average CFR of all the countries we included in the analysis.

### Income level of a country

Countries’ income levels were determined by their Gross National Income (GNI) per capita. We collected the data for 2019 from World Development Indicator on the World Bank website. The income level of a country is determined by the country’s GNI per capita^30^. We also adopted the classification of the income level of countries defined by the World Bank for analysis, which are low-income countries (LICs), lower-middle-income countries (LMICs), higher-middle-income countries (HMICs), and high-income countries (HICs)^31^.

### Human Development Index (HDI)

We used HDI as the indicator to represent countries’ development levels which reflect the social and environmental status of the country^32^. The HDI data was collected from Health Development Report (HDR) 2020 on United Nation Development Program (UNDP) website^33^. The classification of HDI by UNDP was used for analysis in the study. Low development countries are defined as those with index scores below 0.55; while medium, high, and very high development countries whose scores are between 0.55 to 0.69; 0.7 to 0.79; and above 0.8 respectively^32,34^.

### Civil liberties (CL)

While countries’ transparency was reported to be associated with their reported scores by the previous study, we also collected the CL score data from the Freedom House website for analysis^2^. And the category of CL level was also adopted in the study for analysis. “Not-free” countries are those whose score is 0 to 35. While “partially-free” countries and “free” countries are those whose scores are 35 to 70, and above 70 respectively^35–37^.

### Government effectiveness (GE)

The GE is one of the components in The Worldwide Governance Indicators (WGI). It was the indicator reflecting the quality of public services, policy formulation, and its implementation. We chose GE as one of the variables because the literature mentions that the role of government and good governance were very important in efforts to prevent and control infectious diseases^38,39^. The GE data was collected from The WGI project 2020 reports^40^. Since the GE scores range for each country in the report range from -2.5 to 2.5, we classified this variable into 2 categories by setting 0 as the cutoff point. Thus, a country with a GE score above 0 means that the country has a strong GE, and conversely a country with a GE score below 0 is classified as a country with a weak GE.

### Data analysis

196 countries reported their e-SPAR scores in both 2019 and 2020. Among those, only 154 countries with complete data of all indicators were used for analysis. We calculated the countries’ average scores of 11 e-SPAR capacities as well as their average score for each capacity in 2019 and 2020, then calculated their absolute differences in these two consecutive years. In addition, as we found the data were not normally distributed, we conducted the Wilcoxon Sign-rank test for assessing the significance of the difference between scores.

Next, we divided the 154 countries into two groups based on their score classification, namely the group whose scores increased (n = 98), and the group whose scores did not increase (n = 56) for further analysis. A chi-square test was applied to identify the independence of countries' e-SPAR scores to their income levels, HDI, CL, GE, and COVID-19 CFR. Then we performed a multiple linear regression analysis^41^ to determine what factors were associated with the changes in e-SPAR scores. In the Model, the difference between the average e-SPAR scores in 2020 and 2019 was the dependent variable (Y), while the CFR of COVID-19, HDI, CL, and GE were the independent variables (X). We did not include countries’ income levels in the model due to its significant correlation with the index of HDI. We developed three Models in our multiple linear regression analysis to find out what factors were associated with changes in e-SPAR scores in all 154 countries (Model 1), countries whose scores increased (Model 2), and countries whose scores did not increase (Model 3). In the analysis, we’re looking for the adjusted R^2^ value to represent the proportion of the variance for a dependent variable that's explained by independent variables. We used a p-value less than 0.05 as the threshold of statistical significance to reject the null hypothesis. All analysis was performed using the software SPSS, version 18.

## Data Availability

The datasets used and/or analyzed during the current study are available from the corresponding author upon reasonable request.
